# The Structured Operational Research and Training Initiative for Strengthening Health Systems to Tackle Antimicrobial Resistance and Improve Public Health in Low-and-Middle Income Countries

**DOI:** 10.3390/ijerph19084582

**Published:** 2022-04-11

**Authors:** Rony Zachariah, Alex G. Stewart, Jeremiah M. Chakaya, Roger Teck, Mohammed Ahmed Khogali, Anthony D. Harries, Charlotte Seeley-Musgrave, Thomas Samba, John C. Reeder

**Affiliations:** 1 UNICEF, UNDP, World Bank, WHO Special Programme on Research and Training in Tropical Diseases (TDR), 20, Avenue Appia, 27, 1211 Geneva, Switzerland; khogalim@who.int (M.A.K.); reederj@who.int (J.C.R.); 2 College of Life and Environmental Science, University of Exeter, Exeter EX4 4RJ, UK; dragonsteeth@doctors.org.uk; 3 Department of Medicine, Therapeutics and Dermatology, Kenyatta University, Nairobi 00609, Kenya; chakaya.jm@gmail.com; 4 Department of Clinical Sciences, Liverpool School of Tropical Medicine, Liverpool L3 5QA, UK; 5 Manson Unit, Médecins Sans Frontières (MSF), London EC4A 1AB, UK; roger.teck@london.msf.org; 6 Centre for Operational Research, International Union Against Tuberculosis and Lung Disease (The Union), 2 Rue Jean Lantier, 75001 Paris, France; adharries@theunion.org; 7 Department of Clinical Research, Faculty of Infectious and Tropical Diseases, London School of Hygiene and Tropical Medicine, Keppel Street, London WC1E 7HT, UK; 8 Department of Health and Social Care, Victoria St., Westminster, London SW1H 0EU, UK; charlotte.seeley-musgrave@dhsc.gov.uk; 9 Directorate General of Health, Ministry of Health and Sanitation, Freetown 232, Sierra Leone; ttsamba@yahoo.com

## 1. Introduction

*If research is to have an impact and change health outcomes for the better, the findings of the research should be translated into recommendations and actions that can influence policy and/or practice. SORT IT is invaluable for this purpose. Dr. Thomas Samba, Chief Medical Officer, Ministry of Health and Sanitation, Sierra Leone.*

Operational research (OR) is known as the “science of doing better” and is essential to show what works and what does not work in health systems. It can transformatively improve performance and health outcomes [1,2]. There are important synergies between building operational research capacity close to the demand and supply of health services and strengthening health system resilience [3,4]. Two assessments in 2022 that were led by TDR (the UNICEF/UNDP/World Bank/WHO Special Programme for Research and Training in Tropical Diseases) showed that strengthening the core health research capacity of national health workers helped build country resilience to tackling pandemics, including COVID-19 [5,6]. Over 70% of those trained in operational research applied their acquired research skills in various areas along the frontlines of the COVID-19 response. The key message was that investing in people and in building research capacity ahead of public health emergencies is essential to equip the health system with “the right people in the right place at the right time”.

Conducting OR and building national research capacity is essential to tackle global public health challenges, such as antimicrobial resistance (AMR). OR can shape the road map of effective interventions that are needed to enable health systems to perform. In 2019, an estimated 1.27 million deaths were directly attributable to AMR, and there were 4.95 million AMR associated deaths [7]. This ranks AMR next to HIV and tuberculosis as a global public health problem.

In January 2019, the Government of the United Kingdom of Great Britain and Northern Ireland, represented by its Department of Health and Social Care, through the National Institute of Health Research (NIHR), provided funding for a Structured Operational Research and Training Initiative (SORT IT) for strengthening health systems for tackling AMR (AMR SORT IT).

In this editorial, we provide an overview of the SORT IT approach. We then focus on SORT IT on AMR, highlight what has been achieved to date, and where we would like to go from here.

## 2. The Structured Operational Research and Training Initiative (SORT IT) to Strengthen Health Systems

SORT IT is a global partnership-based initiative coordinated by TDR, and implemented with partners. SORT IT seeks to make countries and institutions “data rich, information rich and action rich” to improve public health [1]. The SORT IT model combines research training and research implementation with a hands-on (learning by doing) approach that empowers health personnel and new trainers. There are integrated performance targets and metrics for accountability [8]. The desired impact is strengthened health systems, better programme performance, and improved public health [1].

### 2.1. The Success

Since its inception in 2010, SORT IT has diversified to cover 25 public health domains, has been scaled up to 93 countries, and has built networks and collaborations involving 64 institutions in low-, middle-, and high-income countries [8]. Seventy percent (70%) of completed research has reported an impact on policy and/or practice [9,10], and 51% of trainees independently conduct research after one training cycle, indicating that capacity is built [11]. To ensure quality of evidence, TDR routinely assesses the quality of reporting of SORT IT publications according to international standards. The last assessment showed that 90% of publications (n-392), involving 72 countries and 24 thematic areas, showed excellent reporting quality [12].

### 2.2. The SORT IT on Antimicrobial Resistance (AMR-SORT IT)

This initiative aims to build sustainable operational research capacity to generate and utilize evidence to tackle the emergence, spread, and health impact of AMR. AMR-SORT IT covers seven countries in Asia (Nepal, Myanmar), Africa (Ghana, Sierra Leone, Uganda) and Latin America (Colombia, Ecuador). Strong engagement is sought early with those expected to use the results, such as WHO country offices, national AMR coordinating committees, and SORT IT partners.

### 2.3. The AMR-SORT IT Cycle

**Figure 1:**
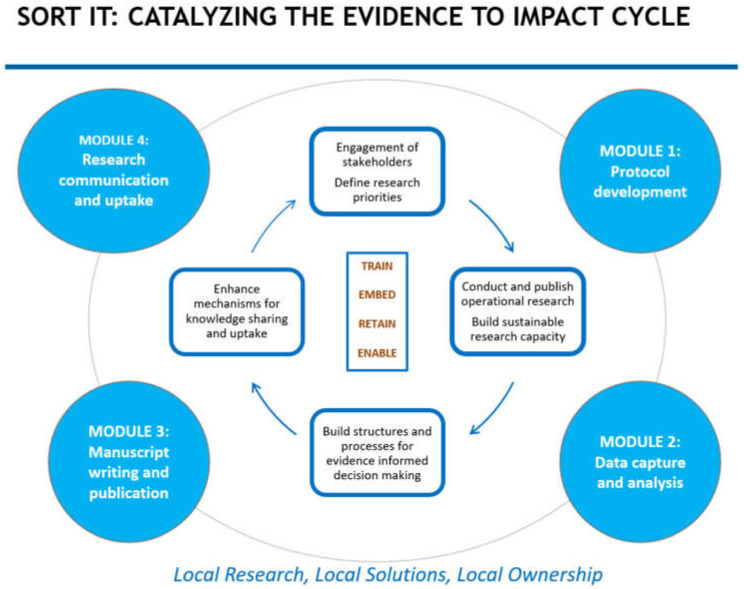
The SORT IT evidence to impact cycle.

The AMR-SORT IT cycle is geared to catalyse the evidence-to-action cycle, from defining the most relevant research in countries to ensuring uptake of research findings (Figure 1). Put simply, AMR SORT IT seeks to conduct “local research for local solutions with local ownership”.

The focus of training is on those who are embedded and retained within health systems. We also seek to enable the structures and processes needed for evidence-informed decision making. SORT IT thus embraces the “Train, Embed, Retain and Enable” strategy for individuals working within health systems which is in line with WHO’s Thirteenth General Programme of Work, 2019–2023 [8].

## 3. Achievements of the SORT IT Project on Antimicrobial Resistance (2019–2022)

The achievements of the SORT IT project can be categorized into: (1) innovating in digital technology—a SORT IT online platform for implementing research during COVID-19; (2) implementing high-quality policy/practice relevant research and building research capacity; (3) strengthening health systems resilience; and (4) building global engagement for AMR through networks and LMIC equitable research partnerships. A brief overview of the main achievements is given below.

*Championed the development and deployment of a “SORT IT online training platform”* to overcome COVID-19 restrictions on travel and gatherings. The SORT IT platform allowed online and “hybrid” training models bringing together individuals from WHO country offices (in 6 countries) and 64 partner institutions to support research studies in Asia, Africa and the Americas (Colombia, Ecuador, Ghana, Myanmar, Nepal, and Uganda).

*Implemented and disseminating high-quality policy/practice relevant research.* To date, 48 research projects have been completed and are being propelled through a novel publication mechanism for timely evidence generation for decision-making. Thirty-five studies from Ghana, Myanmar, Nepal, Sierra Leone, and Uganda have been published in Special Issues of the *Tropical Medicine and Infectious Diseases Journal* [13] and in the *Public Health Action Journal* [14] in a record time of 8–15 weeks. This was achieved by proactively accelerating the journal processes and providing structured support to the researchers, especially by promptly responding to editorial and peer review. Trainees also benefited from a newly developed SORT IT training module on “‘communicating research findings with a KISS—Keep It Short and Simple” [15]. Effective communication of research findings to decision makers is vital for maximizing the opportunities for research uptake.

In terms of capacity building, each research project is used to simultaneously implement four layers of training, involving: (1) frontline workers and programme staff; (2) SORT IT alumni as trainers; (3) academia as trainers; and (4) WHO country staff. On average, three people are trained per research, which adds to the value for money invested.

*Strengthened health systems.* Seventy-three percent (73%) of individuals involved with the SORT IT project continue to apply their early acquired skills in a synergistic manner to the COVID-19 global response. AMR activities contributed to building health system resilience by protecting health workers, keeping health facilities safe, improving laboratory diagnostic capacity, and informing communities on preventive measures.

*Enhanced global engagement through networks to tackle AMR.* The AMR–SORT IT network now includes AMR coordinating committees, WHO regional and country offices, and 64 implementing partners from 26 countries in Europe, Asia, Africa, and the Americas. This is now one of the largest collaborations of institutions involved with operational research in the world.

## 4. Where Do We Go from Here?

Going forward, the main strategic pillars that will guide all project activities include focusing on national priorities; strengthening health systems and research capacity; building a critical mass of trained institutions; and fostering global and community engagement (Box 1).

Box 1 **Main strategic pillars that will guide SORT IT AMR activities in low-and-middle income countries.**

*Operational research that is aligned to country priorities:* We will promote local and nationally relevant research that has the highest likelihood of influencing policy and practice.

*Strengthen health systems and research capacity:* We will build the research capacity of health personnel and implement evidence-based interventions to strengthen the health system. We hope to integrate an organizational culture of research as part of the health system.

*Build a “critical mass” of trained researchers:* Already trained individuals from previous rounds of SORT IT will be actively involved in implementing solutions and training others from the same institutions in conducting new research studies (subsequent rounds of SORT IT). The aim is to create “critical masses” of trained researchers who can conduct and lead further relevant research and provide mentorship. This will allow us to build on what has been already achieved and promote national leadership, sustainability, and greater value for money.

*Global and community engagement:* We will continue to engage with a broad global community and build partnerships and communities of practice to facilitate the role of research for health systems strengthening, and for advocating for the use of high-quality evidence to inform policy and practice. We aim to engage with communities and involve them wherever possible for delivering good and impactful research.

The studies from Sierra Leone in this Special Issue are part of the SORT IT project on tackling AMR and are all identified research priorities that are tailored to national AMR and local needs. They cover environmental issues related to AMR, including household water supply and animal and plant related issues; effects of COVID-19 on AMR; infection prevention and control issues; surgical site infections; the safe use of antimicrobials; and laboratory diagnosis. The next step will be to assess the impact of these studies in informing the decision-making process and strengthening health systems.
